# Amino acid homeostasis and signalling in mammalian cells and organisms

**DOI:** 10.1042/BCJ20160822

**Published:** 2017-05-25

**Authors:** Stefan Bröer, Angelika Bröer

**Affiliations:** Research School of Biology, Australian National University, Linnaeus Way 134, Canberra, ACT 2601, Australia

**Keywords:** amino acid metabolism, amino acid transporters, starvation signalling

## Abstract

Cells have a constant turnover of proteins that recycle most amino acids over time. Net loss is mainly due to amino acid oxidation. Homeostasis is achieved through exchange of essential amino acids with non-essential amino acids and the transfer of amino groups from oxidised amino acids to amino acid biosynthesis. This homeostatic condition is maintained through an active mTORC1 complex. Under amino acid depletion, mTORC1 is inactivated. This increases the breakdown of cellular proteins through autophagy and reduces protein biosynthesis. The general control non-derepressable 2/ATF4 pathway may be activated in addition, resulting in transcription of genes involved in amino acid transport and biosynthesis of non-essential amino acids. Metabolism is autoregulated to minimise oxidation of amino acids. Systemic amino acid levels are also tightly regulated. Food intake briefly increases plasma amino acid levels, which stimulates insulin release and mTOR-dependent protein synthesis in muscle. Excess amino acids are oxidised, resulting in increased urea production. Short-term fasting does not result in depletion of plasma amino acids due to reduced protein synthesis and the onset of autophagy. Owing to the fact that half of all amino acids are essential, reduction in protein synthesis and amino acid oxidation are the only two measures to reduce amino acid demand. Long-term malnutrition causes depletion of plasma amino acids. The CNS appears to generate a protein-specific response upon amino acid depletion, resulting in avoidance of an inadequate diet. High protein levels, in contrast, contribute together with other nutrients to a reduction in food intake.

## Overview

Amino acids are one of the main building blocks of life and are used in a variety of ways by mammalian cells and organisms. (i) Twenty proteinogenic l-amino acids form the building blocks for protein synthesis. (ii) Amino acids are fuels and are metabolised by most cells with a similar energy yield as carbohydrates. (iii) Amino acids are the precursors for many hormones, neurotransmitters and other specialised metabolites such as polyamines, creatine, phosphatidylserine etc. (iv) Amino acids are the principal generators of C1 carbon compounds. (v) Amino acids are important anaplerotic metabolites providing intermediates for the TCA cycle and gluconeogenesis. (vi) Non-essential amino acids can be synthesised by many cells from metabolic intermediates, but essential amino acids must be acquired from nutrients.

While not all of these processes occur at the same time in every cell, this summary illustrates that cells require efficient regulatory mechanisms to ensure a homeostatic intracellular and extracellular amino acid composition. In this review, cellular and systemic amino acid homeostasis will be covered, including how amino acids act as signalling molecules to regulate their diverse functions. In the case of cellular amino acid homeostasis, many examples will be drawn from cancer cells, for which the understanding is more complete than for other cell types. The review of systemic amino acid homeostasis will focus on organs and mechanisms that influence plasma amino acid concentrations.

Before amino acid homeostasis can be considered, it is essential to briefly summarise the mechanisms by which amino acids can signal within cells or whole organisms. It is impossible to cite all original articles in this area and, as a result, emphasis will be given to specialised reviews and recent articles in each area. The authors apologise for any oversight or omission of many important studies in this area.

## Elements of amino acid signalling

The main conduits of amino acid signalling are amino acid-binding proteins, including enzymes and transporters, and tRNA molecules. Binding of amino acids or lack of binding triggers signal transduction events. Most of these events can be understood in terms of amino acid homeostasis, i.e. the provision of an intracellular or plasma amino acid pool for protein biosynthesis and metabolism.

### mTOR

The mTOR pathway — more precisely the mTORC1 complex — is the most well-known amino acid sensor [1–5]. Through its downstream effector p70S6 kinase and direct target 4E-binding protein 1 (4E-BP1), it regulates protein translation, while, through interaction with the ulk1 (UNC51-like kinase 1)/atg13 (autophagy 13)/FIP200 (focal adhesion kinase-interacting protein 200 kDa) complex, it regulates autophagy [6–8]. In general, an active mTORC1 complex will promote protein biosynthesis and subdue autophagy, while an inactive mTORC1 complex does the opposite.

**Figure 1.**
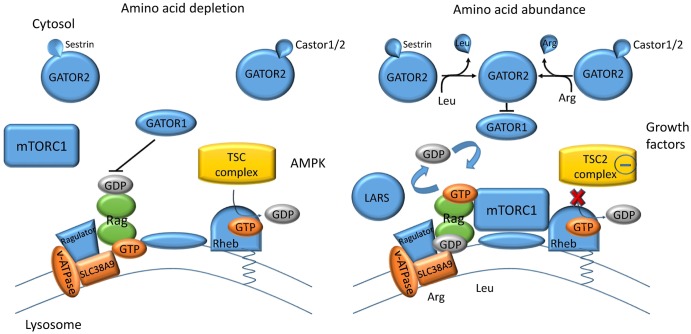
Amino acid sensing by the mTORC1 complex. When amino acids are depleted, mTORC1 is located in the cytosol and inactive. Energy depletion can also switch off mTORC1 signalling by converting Rheb into its GDP-bound form. In the presence of growth factors, TSC2 is inactive and mTORC1 binds to GTP-bound Rheb at the surface of lysosomes. Cytosolic amino acids such as leucine and arginine can activate mTORC1 by inhibiting GATOR1. This switches the GTP/GDP-bound state of Rag proteins (green) and activates mTORC1. In the lysosome, arginine binds to transceptor SLC38A9, which activates mTORC1 through associated proteins. Some parts of the model are not universally accepted (for details see text).

It is noteworthy that mTORC1 integrates many signals through it being regulated by the Tuberous sclerosis complex 2 (TSC2) [9]. For instance, mTORC1 requires indirect activation through protein kinase AKT (growth factors) or ERK (mitogens), which in turn phosphorylates and inactivates TSC2 [10]. The TSC2 subunit has GTPase activity towards mTORC1 activator Rheb (Ras homologue enriched in brain), thereby inactivating Rheb by hydrolysing its GTP ligand to GDP (Figure 1). Thus, mTORC1 can only be activated by amino acids as long as TSC2 remains inactive. In addition to lack of the inactivating signals, TSC2 can be activated through AMPK, thus switching mTORC1 off when energy is depleted [11,12].

The main amino acids that activate mTORC1 are leucine and arginine [1,2]. The mTORC1 complex has sensors that sample the cytosol and the lumen of the lysosome for amino acids (Figure 1). The precise mechanisms of amino acid sensing by mTORC1 are just beginning to emerge. When cells are deprived of amino acids, mTORC1 is found throughout the cytoplasm. Upon the addition of amino acids, mTORC1 rapidly translocates to the lysosomal surface, where it is activated by Rheb [13]. Other relevant elements are the vacuolar H^+^-ATPase, the ragulator complex Rag GTPases (Rag A/B and C/D) and the lysosomal arginine transceptor (definition see below) SLC38A9. This transceptor appears to be the lysosomal arginine sensor [14–16]. In support of the notion of lysosomal amino acid sensing, depletion of lysosomal amino acids through overexpression of the lysosomal proton amino acid transporter PAT1 (SLC38A1) turns off mTORC1 signalling [17]. Loading of lysosomes with leucine occurs through proteolysis, but is also mediated by the heteromeric transporter 4F2hc-LAT1 (see below), which is recruited to the lysosomal membrane by the LAPTM4b protein [18]. A lysosomal leucine sensor has not been identified though. Cytosolic amino acid levels are thought to change the nucleotide-bound state of Rag GTPases associated with mTORC1. Starving induces a GDP-bound state, which is quickly changed to a GTP-bound state when amino acid levels are sufficient. The exchange between GDP and GTP appears to be regulated by associated proteins directly involved in amino acid sensing. Castor1 homodimers or Castor1/2 heterodimers act as cytosolic arginine-binding proteins to activate mTORC1 [19]. The activation is mediated through GATOR1 and/or GATOR2. The GATOR1 complex, in turn, inhibits mTORC1 activity by hydrolysing Rag-bound GTP to GDP. Sestrin2 is thought to act as a cytosolic leucine-binding protein, also regulating mTORC1 via GATOR1 and/or 2 [20,21]. Some aspects of this model are still disputed and alternative models have been put forward [3,22]. Most notably, leucyl-tRNA synthetase (LARS) has been suggested as the cytosolic leucine sensor for mTORC1 acting as a GTPase-activating protein (GAP) for RagD [23]. In another variation, sestrin's role as a possible leucine sensor has been questioned [24] because of its multiple roles in cellular stress signalling and because yeast mTORC1 appears to sense leucine without sestrin isoforms. The role of transporters/transceptors in this model is also still unclear. Based on work in *Drosophila* and later in cancer cells, Goberdhan's group [3,25] found that lysosomal proton amino acid transporters 1 and 4 (PAT1/4) were required for amino acid signalling by mTORC1, not turning it off.

### GCN2 (general control non-derepressable 2)/uncharged-tRNAs

**Figure 2.**
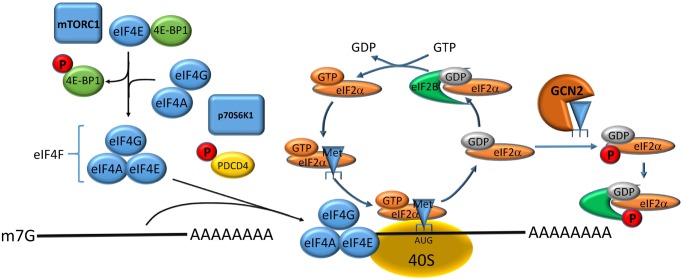
Regulation of protein translation at the initiation stage. To initiate translation, the capped (m7G) mRNA has to form a complex with eIF4A/G/E (termed eIF4F), which is facilitated by phosphorylation of the eIF4E-binding protein 4E-BP1 through the action of mTORC1. This complex binds to the 40S ribosomal subunit, which starts scanning the mRNA until it reaches the start cordon. At this point eIF2α·GTP·Met-tRNA_i_^Met^ can bind to the start codon, resulting in formation of the full ribosome and start of translation. In this process, eIF2α·GTP is hydrolysed to eIF2α·GDP, which needs to be recharged to eIF2α·GTP through the action of eIF2B to initiate another translation event. When GCN2 is activated by uncharged tRNAs, it phosphorylates eIF2α, thereby inhibiting the exchange from GDP back to GTP.

While mTORC1 responses are optimised to sense amino acid sufficiency, the GCN2 (general control non-derepressable 2)/ATF4 system in mammalian cells has evolved to sense amino acid restriction or more precisely amino acid imbalance [26–28]. Its main consequences are to reduce global translation and at the same time to increase the cellular amino acid pool through increasing biosynthesis and amino acid transport (Figure 2). Any depletion of a particular amino acid will eventually result in unloaded tRNAs. Owing to degeneracy of the genetic code, several isoacceptor-tRNAs exist for most amino acids; these compete with each other for the same amino acids in the cytosol [29]. The amount of uncharged tRNA depends on the supply of a specific tRNA resulting from unloading its cognate amino acid at the ribosome and the demand by the competing aminoacyl-tRNA synthetases. It has been shown in *Escherichia coli* and in yeast that reduction in the amino acid supply quickly results in the appearance of uncharged tRNAs, particularly of frequently used codons [29]. Uncharged tRNAs are important signalling molecules as they can bind to and activate protein kinase GCN2. The target protein for activated GCN2 is the eukaryotic initiation factor 2α (eIF2α), which can also be phosphorylated by other stress-activated protein kinases. In all cases, initiation of translation is reduced, thereby reducing the demand for amino acids. GCN2 consists of several domains [30], of which the histidyl-tRNA synthetase-like domain is thought to provide the binding site for uncharged tRNAs, which activates the protein kinase domain. Uncharged tRNAs thus serve as surrogate measures for amino acid depletion. While the vast majority of mRNAs are translated less when GCN2 is activated, specific mRNAs containing upstream open reading frames (uORFs) are actually translated more efficiently, resulting in the production of transcription factor ATF4, which in turn has binding sites in the regulatory region of many genes that restore amino acid levels such as amino acid transporters and enzymes involved in amino acid biosynthesis (outlined in more detail below).

### G-protein-coupled receptors

Several class C G-protein-coupled receptors (GPRs) have been identified as amino acid receptors [31,32]. In this family, the metabotropic glutamate receptors respond specifically to glutamate, modulating neurotransmission on a slower time scale than the fast glutamate-gated ion channels [33]. The T1R1/T1R3 taste receptor, the calcium receptor and GPRC6A, in contrast, respond to a variety of amino acids [32]. More recently, GPR142 has been identified as a sensor for aromatic amino acids [34]. These receptors have a more systemic role in amino acid homeostasis, regulating food intake and hormone secretion, particularly in the intestine [35,36]. Taste receptors have been shown to bind amino acids and appear to regulate many elements in amino acid homeostasis. Increased expression of transcription factor ATF2 — which is required for the amino acid depletion responses of genes such as ATF3, CHOP, SARS and 4EBP-1 — is mediated by a signal transduction pathway that appears to involve an amino acid sensing GPR [37].

### Transceptors

Transporters go through a cycle of conformational changes during transport, which could be used to convey signals about nutrient abundance, similar to GPR. However, this mode of direct signalling is difficult to discriminate from indirect signalling by the transported amino acids through conduits such as mTORC1 or GCN2. A dual mode of transport and signalling has been termed transceptor [38–40]. The best evidence for transceptor function in mammalian cells comes from adaptive regulation of SNAT2 [41]. This transporter is expressed at low levels in complete media, but its activity is induced many-fold by amino acid depletion [42]. The regulation is complex as it involves increased transcription [43], increased protein translation [42], increased mRNA stability and reduced protein degradation [44]. Most of these functions can be suppressed by the addition of substrates of the transporter, suggesting a direct signalling function of the transporter. Some of these effects are mediated via GCN2, but also the substrate analogue *N*-methyl-aminoisobuyric acid prevents expression, although it is transported very slowly and not used for protein biosynthesis. Another example of a transceptor is SLC38A9, which mediates lysosomal arginine signalling [14,15], but also has transport function. However, the release of arginine into the cytosol is unlikely to provide this signal. How SNAT2 and SLC38A9 transmit signals remains unclear.

### Allosteric enzymes

An important aspect of amino acid signalling is allosteric regulation of metabolic enzymes by amino acids or their metabolites. The most well-known examples in mammalian cells are carbamoylphosphate synthetase (CPS1) [45] and glutamate dehydrogenase (GDH) [46]. CPS1 controls the entry of amino groups into the urea cycle and adjusts urea production to the prevalence of glutamate and arginine in the liver [47]. Glutamate dehydrogenase is regulated by leucine, either regulating glutamate levels and urea cycle activity in the liver [48] or regulating the entry of glutamate into the TCA cycle [49].

## Cellular amino acid homeostasis

Several factors regulate amino acid homeostasis in cells: (i) entry and exit through amino acid transporters; (ii) amino acid biosynthesis and degradation and (iii) protein biosynthesis and degradation.

### Entry and exit through amino acid transporters

**Figure 3.**
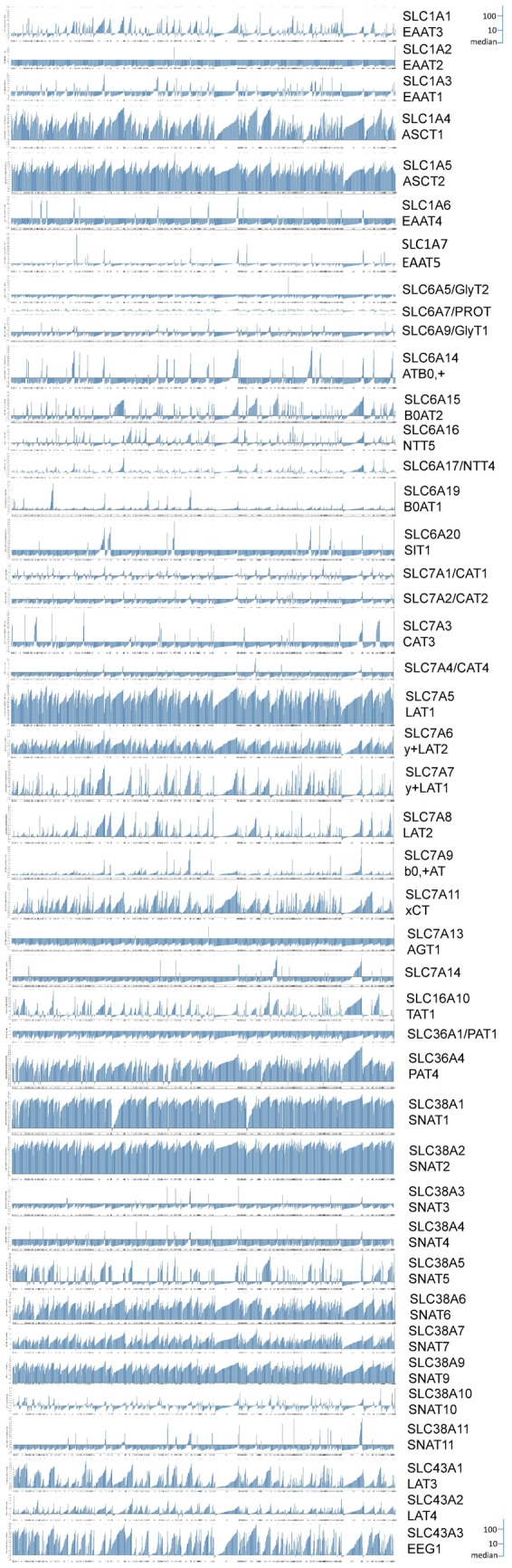
The repertoire of amino acid transporters in cancer cells. In rapidly growing cancer cells, certain amino acid transporters are prevalent. Amino acid transporter transcript levels were analysed through Oncomine in 917 cell lines of the Barretina dataset [58] and are depicted as vertical blue bars. The dataset is subdivided by vertical lines into different groups of cancers. Data are depicted as log2 expression levels relative to the median expression of all genes in the genome. The size of each panel was adjusted to fit the scale. Blue-coloured bars pointing upwards from the median indicate well-expressed genes; bars pointing downwards indicate weakly expressed genes. Only a handful of transporter genes show high expression in essentially all cell lines (upward-pointing blue bars across the complete *x*-axis). These transporters are likely to be essential for amino acid homeostasis, whereas other transporters occur only in selected cancer cell lines and are likely to have specialised roles.

**Figure 4.**
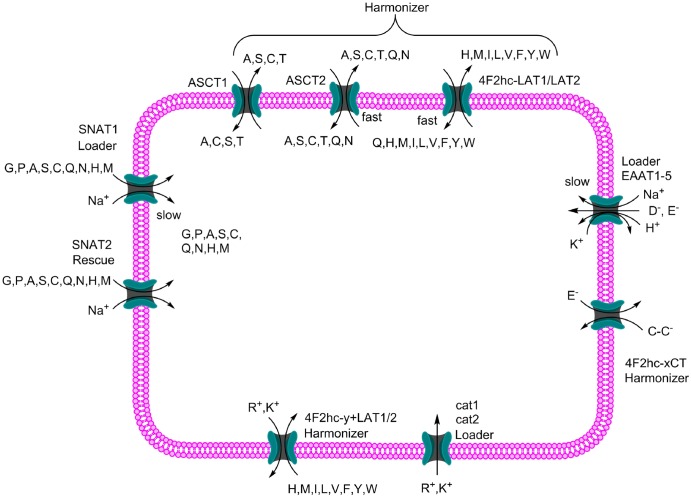
A general model of amino acid accumulation in non-epithelial cells. The model makes use of amino acid loaders, which accumulate a certain group of amino acids in the cytosol. Mostly, the Na^+^-electrochemical gradient is used to actively transport amino acids. A second group of transporters are the harmonisers, which exchange one amino acid for another and have overlapping substrates with amino acid loaders. The exchange mechanism ensures that depletion of a particular amino acid is avoided. Under conditions of amino acid depletion, rescue transporters are up-regulated to bring in more amino acids.

The study of amino acid transporters in mammalian cells was pioneered by Halvor Christensen [50], who recognised that certain groups of amino acids compete with each other for uptake into cells. More detailed studies revealed many transport activities, which were subsequently confirmed by molecular cloning, although significant refinements became obvious through molecular identification [51–53]. The known amino acid transporters and their main properties are listed in Table 1. One of the more puzzling findings emanating from molecular cloning and characterisation of amino acid transporters was the prevalence of amino acid transporters that mediate obligatory amino acid exchange instead of net transport mechanisms, members of which are abundant in many cell types. This is in contrast with the transport of glucose, which has served as a model for metabolite homeostasis for many years [54]. Glucose is actively accumulated by Na^+^–glucose co-transporters, which use the electrochemical gradient of Na^+^ to drive accumulation of glucose inside the cell, most notably in epithelial cells of the intestine and kidney. Once accumulated in the intestinal and renal epithelium, glucose is then distributed along its concentration gradient through uniporters (also known as facilitated diffusion) ensuring supply of glucose to all cells in the body. This simple and effective system is only partially applicable to amino acids, mainly because a pool of all 20 proteinogenic amino acids must be maintained in the cytosol. In contrast, mammalian cells do not maintain a significant pool of free glucose in the cytosol, due to the presence of hexokinase, which immediately converts glucose into glucose-6-phosphate. The early glycolytic intermediates glucose-6-phosphate and fructose-6-phophate rather form a metabolic pool, which can be used in different metabolic pathways. In the case of amino acids, cells must ensure a homeostatic pool of the 20 proteinogenic amino acids inside the cell to charge tRNA molecules for protein biosynthesis. Accumulation of uncharged tRNAs causes a stress response as outlined above. Moreover, amino acid concentrations in the cytosol are significantly higher than in blood [55,56]; uniporters (facilitated diffusion) would therefore reduce intracellular amino acid concentrations. The human genome contains ∼50 different amino acid transporters, many of which carry out specialised roles in selected cell types [51,57]. To identify a minimal set of transporters required for cellular amino acid homeostasis, the expression of all known plasma and lysosomal amino acid transporters is shown for a set of 917 cancer cell lines (Figure 3) [58]. Consistently and highly expressed amino acid transporters are: ASCT1, ASCT2, LAT1, y^+^LAT2, PAT4 (lysosomal), SNAT1, SNAT2, SNAT6, SNAT7 and SLC38A9 (lysosomal). This mixture of antiporters and Na^+^-dependent symporters can be combined to generate a pool of elevated amino acid concentrations in the cytosol (Figure 4). To illustrate the mechanism of amino acid homeostasis in more detail, the example of 143B osteosarcoma cells is used [59], which express such a simple set of amino acid transporters. However, a similar set of transporters is expressed in many cancer cell lines (Figure 3) [58] and also in muscle (see below). In 143B cells, Na^+^-neutral amino acid symporter 1 (SNAT1) mediates the transport of small hydrophilic neutral amino acids and accumulates them in the cytosol (Figure 4). This transporter is designated as a ‘loader’. Once loaded into the cytosol, these amino acids are used as exchange currency to mediate the uptake of other small neutral amino acids through amino acid exchangers ASCT1 [60] or ASCT2 [61]. Exchangers are also used to mediate the uptake of large branched-chain and aromatic amino acids through the action of heteromeric amino acid exchangers 4F2hc-LAT1 [62,63] and 4F2hc-LAT2 [64,65]. There needs to be an overlap of substrate specificity between loaders and exchangers. The amino acid exchangers dominate when transport is analysed using radiolabelled amino acids, demonstrating that exchange processes are faster than the net uptake mediated by Na^+^-neutral amino acid symporters [59]. This ensures a harmonised mixture of all 20 amino acids. For instance, if leucine depletes inside the cell through protein biosynthesis and metabolism, the chances of this amino acid being transported out of the cell are minimal, while the chances to be imported against an abundant intracellular amino acid are much higher. As a result, the pool of leucine will be automatically restored. These transporters are designated ‘harmonisers’ because they automatically restore a harmonised mixture of all transported substrates. Three amino acids are more abundant in blood plasma than other amino acids, namely, glycine, alanine and glutamine. All of these are substrates of Na^+^-neutral amino acid symporters, such as SNAT1, and are accumulated to even higher concentrations within the cell, where they serve as exchange currency to drive the uptake of other amino acids [66–68]. Glycine, alanine and glutamine are also non-essential amino acids and can be synthesised within the cell for exchange purpose. Other amino acids may also serve as exchange substrates, e.g. asparagine [69]. Supporting this notion, synthesis of most non-essential amino acids is induced upon amino acid restriction [70]. Amino acid restriction also causes up-regulation of transporters such as SNAT2, which will help in loading amino acids into the cytosol (designated as rescue transporters) [42]. It is interesting to note that lysosomal amino acid transporter PAT1, the overexpression of which switches off mTORC1 signalling, is expressed at very low levels in cancer cells (Figure 3).

**Table 1:** Amino acid and peptide transporters and their properties

SLC	Acronym	Substrates	Function	Mechanism	Loc
SLC1A1	EAAT3	D,E,Cn	System X^−^_AG_	S: 3Na^+^/1H^+^ A:1K^+^	PM
SLC1A2	EAAT2	D,E	System X^−^_AG_	S: 3Na^+^/1H^+^ A:1K^+^	PM
SLC1A3	EAAT1	D,E	System X^−^_AG_	S: 3Na^+^/1H^+^ A:1K^+^	PM
SLC1A4	ASCT1	A,S,C	System ASC	Antiporter	PM
SLC1A5	ASCT2	A,S,C,T,Q	System ASC	Antiporter	PM
SLC1A6	EAAT4	D,E	System X^−^_AG_	S: 3Na^+^/1H^+^ A:1K^+^	PM
SLC1A7	EAAT5	D,E	System X^−^_AG_	S: 3Na^+^/1H^+^ A:1K^+^	PM
SLC3A1	rBAT	Trafficking	Heavy chains of		PM
SLC3A2	4F2hc	subunits	Heteromeric AA Transporter		PM
SLC6A5	GlyT2	G	System Gly	S: 3Na^+^/1Cl^−^	PM
SLC6A7	PROT	P	Proline transporter	S: 2Na^+^/1Cl^−^	V
SLC6A9	GlyT1	G	System Gly	S: 2Na^+^/1Cl^−^	PM
SLC6A14	ATB^0,+^	All neutral and cationic	System B^0,+^	S: 2Na^+^/1Cl^−^	PM
SLC6A15	B^0^AT2	P,L,V,I,M	System B^0^	S: 1Na^+^	PM
SLC6A17	NTT4/B^0^AT3	L,M,P,C,A,Q,S,H,G	System B^0^	S: 2Na^+^/1Cl^−^	PM/V
SLC6A18	XT2/B^0^AT3	G, A	System Gly	S: 2Na^+^/1Cl^−^	PM
SLC6A19	B^0^AT1	All neutral	System B^0^	S: 1Na^+^	PM
SLC6A20	SIT1	P	System IMINO	S: 2Na^+^/1Cl^−^	PM
SLC7A1	CAT-1	K,R,O	System y^+^	Uniporter	PM
SLC7A2	CAT-2	K,R,O	System y^+^	Uniporter	PM
SLC7A3	CAT-3	K,R,O	System y^+^	Uniporter	PM
SLC7A4	CAT-4	Unknown	Unknown	Unknown	PM
SLC7A5	LAT1/4F2hc	H,M,L,I,V,F,Y,W	System L	Antiporter	PM
SLC7A6	y^+^LAT2/4F2hc	K,R,Q,H,M,L	System y^+^L	S:AA^0^-Na^+^ A:AA^+^	PM
SLC7A7	y^+^LAT1/4F2hc	K,R,Q,H,M,L,A,C	System y^+^L	S:AA^0^-Na^+^ A:AA^+^	PM
SLC7A8	LAT2/4F2hc	All neutral except P	System L	Antiporter	PM
SLC7A9	b^0,+^AT/rBAT	R,K,O,Cn	System b^0,+^	Antiporter	PM
SLC7A10	Asc-1/4F2hc	G,A,S,C,T	System asc	Antiporter	PM
SLC7A11	xCT/4F2hc	D,E,Cn	Sytem x^−^_c_	Antiporter	PM
SLC7A12	Asc-2	G,A,S,C,T	System asc	Antiporter	PM
SLC7A13	AGT1/rBAT	Cn	Renal cystine transporter	Antiporter	PM
SLC7A14		K,R,O	System c	Uniporter	L
SLC15A1	PEPT1	Di-, Tri-peptides	Intestinal peptide transporter	S: H^+^	PM
SLC15A2	PEPT2	Di-, Tri-peptides	Renal peptide transporter	S: H^+^	PM
SLC15A3	PHT2	Di-, Tri-peptides	Lysosomal peptide transporter	S: H^+^	L
SLC15A4	PHT1	Di-, Tri-peptides	Lysosomal peptide transporter	S: H^+^	L
SLC16A10	TAT1	W,Y,F	System T	Uniporter	PM
SLC17A6	VGLUT2	E	Vesicular Glu transporter	Uniporter	V
SLC17A7	VGLUT1	E	Vesicular Glu transporter	Uniporter	V
SLC17A8	VGLUT3	E	Vesicular Glu transporter	Uniporter	V
SLC25A2	ORC2	K,R,H,O,Cit	Orn/Cit carrier	Antiporter	M
SLC25A12	AGC1	D,E	Asp/Glu carrier	Antiporter	M
SLC25A13	AGC2	D,E	Asp/Glu carrier	Antiporter	M
SLC25A15	ORC1	K,R,H,O,Cit	Orn/Cit carrier	Antiporter	M
SLC25A18	GC2	E	Glu carrier	A: OH^−^	M
SLC25A22	GC1	E	Glu carrier	A: OH^−^	M
SLC32A1	VIAAT	G,GABA	Vesicular Gly/GABA transporter	A: H^+^	V
SLC36A1	PAT1	G,P,A	Proton amino acid transporter	S: H^+^	PM,L
SLC36A2	PAT2	G,P,A	Proton amino acid transporter	S: H^+^	PM
SLC36A3	PAT3	Unknown	Unknown	Unknown	PM
SLC36A4	PAT4	P,W	Amino acid sensor	Unknown	PM,L
SLC38A1	SNAT1	G,A,N,C,Q, H,M	System A	S:Na^+^	PM
SLC38A2	SNAT2	G,P,A,S,C,Q,N,H,M	System A	S:Na^+^	PM
SLC38A3	SNAT3	Q,N,H	System N	S:Na^+^ A:H^+^	PM
SLC38A4	SNAT4	G,A,S,C,Q,N,M	System A	S:Na^+^	PM
SLC38A5	SNAT5	Q,N,H,A	System N	S:Na^+^ A:H^+^	PM
SLC38A6	SNAT6	Unknown	Unknown	Unknown	PM
SLC38A7	SNAT7	Q,N,H,A,S,D	Unknown	S:Na^+^	PM
SLC38A8	SNAT8	Q,A,R,H,D	System A	S:Na^+^	PM
SLC38A9		Q,R,N	Lysosomal transporter	S:Na^+^	L
SLC38A10		Unknown	Unknown	Unknown	
SLC38A11		Unknown	Unknown	Unknown	
SLC43A1	LAT3	L,I,M,F,V	System L	Uniporter	PM
SLC43A2	LAT4	L,I,M,F,V	System L	Uniporter	PM
CTNS	Cystinosin	Cn	Lysosomal transporter	S: H^+^	L

Substrates are given in one letter code: Cn, cystine; O, ornithine; Cit, citrulline; GABA, γ-aminobutyric acid. The column ‘function’ includes reference to amino acid transport systems. These systems have acronyms indicating the substrate specificity of the transporter. Upper case symbols indicate Na^+^-dependent transporters (with the exception of system L, T and the proton amino acid transporters); lower case is used for Na^+^-independent transporters (for example asc, y^+^ and x^−^_c_). Letters X^−^ or x^−^ indicate transporters for anionic amino acids (as in X^−^_AG_ and x^−^_c_). The subscript AG indicates that the transporter accepts aspartate and glutamate; the subscript c indicates that the transporter also accepts cystine. Letter y^+^ refers to Na^+^-independent transporters for cationic amino acids; B or b refers to amino acid transporters of broad specificity with superscript ‘0’ indicating a transporter accepting neutral amino acids and superscript ‘+’ indicating a transporter for cationic amino acids. T stands for a transporter for aromatic amino acids, and system N indicates selectivity for amino acids with nitrogen atoms in the side-chain. In the remaining cases, the preferred substrate is indicated by the one letter code for amino acids. For example, system L refers to a leucine preferring transporter and system ASC refers to a transporter preferring alanine, serine and cysteine. Proline and hydroxyproline are referred to as imino acids. Due to historic idiosyncrasies, the nomenclature for plasma membrane amino acid transport systems is not completely consistent, but widely used in the field. Column mechanism: S, symport; A, antiport. Column localisation: PM, plasma membrane; V, vesicular; L, lysosomal; M, mitochondrial.

References for transporter families: SLC1 [239]; SLC3 and SLC7 [171,240]; SLC6 [241,242]; SLC16A10 [243]; SLC15 [174]; SLC17 [244]; SLC25 [245]; SLC32 [246]; SLC36 [247]; SLC38 [66,67,248,249]; SLC43 [250]; CTNS [251].

The homeostasis of cationic amino acids is slightly different because of the positive charge associated with lysine, arginine and ornithine (Figure 4). In non-epithelial cells, cationic amino acid transporters (cat1–3) are expressed that mediate facilitated diffusion [71]. However, facilitated diffusion is accumulative due to the inside-negative membrane potential of mammalian cells [72]. It is noteworthy that cationic amino acid transporters can also mediate amino acid exchange and are up-regulated under amino acid deprivation [73]. As a result, they can serve as a loader, harmoniser and rescue in one molecule. Even for cationic amino acids, many cells express heteromeric antiporters such as 4F2hc-y^+^LAT2, which mediates the efflux of cationic amino acids in exchange for neutral amino acids plus Na^+^ [74]. Owing to the concentration gradient of Na^+^, a flux of neutral amino acids into the cytosol is favoured. The positive charge of Na^+^ is balanced by the efflux of the cationic amino acids. Thus, it appears that cellular levels of cationic amino acids are balanced by net import through cat1–3, net export through 4F2hc-y^+^LAT2 and metabolism (Figure 4).

Anionic amino acids would normally be extruded from mammalian cells due to their negative charge. This can occur under extreme conditions as a rescue mechanism, such as cell swelling [75]. Under physiological conditions, anionic amino acid transport occurs in symport with 3 Na^+^ ions and 1 H^+^, and in exchange for 1K^+^ (Figure 4) [76]. This mechanism not only compensates for the negative charges of glutamate and aspartate, but also provides extensive additional driving force to accumulate anionic amino acids in the cytosol, potentially up to one million-fold. The mechanism is particularly important in the brain, where neurotransmitter glutamate has to be cleared from the extracellular space to sub-micromolar levels [77]. However, in most other cells, the strong accumulative power of glutamate transporters carries the risk of cell swelling due to excessive accumulation of glutamate. Aspartate is not very abundant in blood plasma, but glutamate concentration can be as high as 100 μM. As a result, glutamate is the prominent amino acid in many cells, but its concentration is in the 5–10 mM range, equating to a modest 100-fold accumulation. Four mechanisms avoid excessive accumulation of glutamate in the cytosol. First, Na^+^-dependent glutamate transporters (SLC1 family) show a strong tendency for exchange, which increases with intracellular substrate accumulation [78]. Second, apart from astrocytes in the brain, glutamate transporter expression is rather low (e.g. Figure 3). Third, glutamate can leave the cell through amino acid exchangers and non-specific anion transporters. Notably, 4F2hc-xCT, which exchanges intracellular glutamate for extracellular cystine^−^ [79], is abundant in many cancer cells. Cystine in turn is immediately reduced into cysteine inside the cytosol. This is an important mechanism for maintenance of glutathione and provides a constant leak pathway for glutamate out of the cell [80]. Fourth, glutamate is actively metabolised through the TCA cycle. Thus it appears that glutamate equilibrium in cells is determined by influx through Na^+^-dependent glutamate transporters (SLC1 family), which is quickly limited by exchange, efflux through pathways such as the heteromeric glutamate-cystine exchanger 4F2hc-xCT and removal through metabolism.

### Biosynthesis and degradation

*Biosynthesis:* Non-essential amino acids can be synthesised by a variety of cells. Not surprisingly, many pathways are activated by amino acid starvation, notably asparagine synthetase (ASNS), which generates asparagine from aspartate using glutamine as the amino group donor [27]. Except pancreas, most tissues express low levels of the enzyme, but its expression increases significantly under amino acid limitation. This is mediated by two nutrient response elements in the promoter of the ASNS gene [27]. Initially, an amino acid response element (AARE) was detected at position −68/−60 and later another nutrient response element (CARE) was located at position −48/−40 [CARE: CCAAT/enhancer-binding protein (C/EBP)-–ATF response element]. Within 45 min of amino acid depletion, newly synthesised transcription factor ATF4 binds to the CARE region and this initiates transcription of ASNS [81]. The generation of asparagine under conditions of amino acid depletion also makes sense in conjunction with the set of transporters required for amino acid homeostasis described above. Asparagine is a substrate for ASCT2, where it can serve as an exchange substrate to bring in other amino acids required for cell growth [69]. Translation of ATF4 itself is regulated by amino acid availability through an uORF-regulated translational mechanism described below. ATF4-binding sites are found in promoters of genes encoding biosynthetic enzymes of most non-essential amino acids and many amino acid transporters [82,83]. In the case of proline, it was demonstrated that intracellular proline concentration increased in an ATF4-dependent manner [84–86]. Similar to asparagine and serine, it is not an essential amino acid, but substantial carbon flow from glutamine is diverted into proline synthesis in rapidly proliferating cells. The reason for this metabolic flux remains largely unknown, but the special role of proline, for example for the maintenance of stem cells, is well established [86,87].

Many cancer cells express high levels of phosphoserine aminotransferase 1 (PSAT1) [88,89]. In these cells, 3-phosphopyruvate is an important acceptor for amino groups producing phosphoserine, which is then converted into serine. The amino groups, at least in part, are derived from glutamate, which in the process is converted into 2-oxoglutarate providing TCA cycle intermediates [90]. 3-Phosphopyruvate is produced from glycolytic intermediate 3-phosphoglycerate. Serine is an important generator of C1 groups in many cells resulting in the generation of glycine [88]. Serine biosynthesis is also regulated by the transcription factor ATF4 and its interacting partner nuclear factor erythroid-2-related factor 2 (NRF2) [91]. Incidentally, serine is also an exchange substrate for ASCT2 and may serve a similar role to recruit other amino acids through exchange processes.

Aminotransferases hold an important role in amino acid homeostasis, because *de novo* synthesis of amino acids from organic acids requires the transfer of an amino group, which has to be derived from another amino acid [92]. Some enzymes, such as glutamine synthetase and GDH, can use ammonium ions (NH_4_^+^), which are ultimately also derived from other amino acids.

In summary, biosynthesis of non-essential amino acids is an active process in many cell types and contributes to the maintenance of intracellular amino acid levels. Almost all pathways are up-regulated by transcription factor ATF4, which is induced upon amino acid limitation.

**Figure 5.**
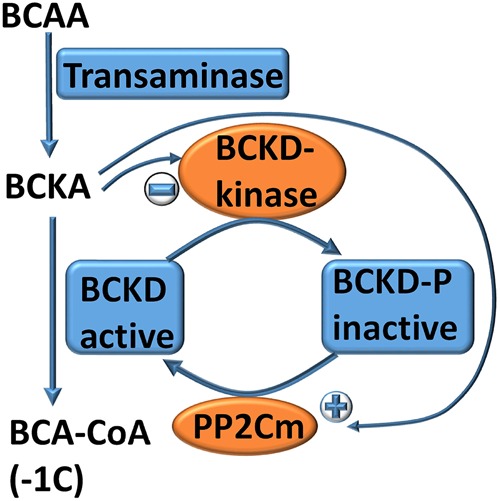
Autoregulation of amino acid metabolism. In the first step of their metabolism, BCAAs are converted into the corresponding keto-acids through BCAA transaminase. The second step is the oxidative decarboxylation into coenzyme A thioesters of branched organic acids via BCAA dehydrogenase (BCKD). Alpha-ketoisocaproate is generated from leucine and is a potent activator of protein phosphatase PP2Cm and a potent inhibitor of BCKD kinase. The two actions dephosphorylate BCKD to be active. Depletion of BCAAs will result in the cessation of metabolism through the reverse events.

*Degradation:* Amino acids can be oxidised in many cells and are important anaplerotic substrates for TCA cycle intermediates. Oxidation takes place in the mitochondria, but the transport mechanism for most amino acids into mitochondria is unknown. Almost all cells are able to oxidise branched-chain amino acids (BCAAs), but must balance the use of these essential amino acids for anaplerosis, energy generation and protein biosynthesis to avoid depletion [93,94]. The metabolism of the three BCAAs, leucine, isoleucine and valine, is regulated co-ordinately through the branched-chain keto-acid dehydrogenase (BCKD) complex, the second step in the breakdown pathway for BCAAs (Figure 5) [95]. This complex is analogous to the pyruvate dehydrogenase complex, catalysing the oxidative decarboxylation of branched-chain keto-acids to CoA-derivatives of the one-carbon shorter organic acid. BCKD is tightly controlled through phosphorylation of its E1 subunit by BCKD kinase and dephosphorylation by the protein phosphatase PP2Cm (Figure 5) [96]. In the first step of metabolism, BCAAs are transaminated into the corresponding branched-chain keto-acids (BCKAs). Interestingly, mitochondrial BCAA transaminase (BCAT2) has an ATF4-binding site in its promoter, suggesting that BCAAs can serve as amino group donors for the synthesis of non-essential amino acids. The basal expression level of BCAT2 is high; as a result, levels of BCKA reflect the abundance of BCAAs. α-Ketoisocaproate, produced from leucine, is a key allosteric regulator of BCKD kinase and PP2Cm in opposite directions. It activates PP2Cm and inhibits BCKD kinase. This ensures that excess BCAAs are oxidised, but any depletion will quickly result in cessation of metabolism. Leucine is the most frequently used amino acid for protein biosynthesis in humans [97] and the most frequently encoded essential amino acid in a wide variety of animals [98], while cytosolic levels of all BCAAs are similar. Thus, it is likely that leucine is the most sensitive indicator of BCAA depletion.

Another amino acid that is heavily metabolised in proliferating cells is glutamine. Many cancer cells are in fact glutamine-dependent [99,100], for they use glutamine as a precursor for a variety of building blocks, such as amino acids, nucleotides and fatty acids. It is worth noting that glutamine cannot be directly metabolised by the TCA cycle, which only metabolises acetyl-CoA, but not its intermediates. Full oxidation of TCA cycle intermediates requires malic enzyme, which converts malate into pyruvate [101]. Glutamine metabolism in rapidly proliferating cells instead makes use of a linear version of the TCA cycle, aptly named glutaminolysis [102]. This pathway allows glutamine to be converted into a variety of metabolic intermediates such as aspartate, asparagine, citrate and oxaloacetate and pyruvate [103]. In differentiated cells, this pathway is of limited activity. In fact, most differentiated cells use glutamine synthetase to bind excess ammonia to form glutamine [104]. Owing to the abundance of glutamine, it appears unlikely that glutamine would be easily depleted in mammalian cells. As pointed out above, it rather serves a role as an exchange currency to bring in other amino acids. Glutaminase is a highly regulated enzyme. Cells that express high levels of the transcription factor myc up-regulate glutaminase expression more than 10-fold [105]. This is not mediated by direct transcriptional control of the glutaminase promoter, but rather by increasing mRNA stability through repression of micro-RNA miR-23. In this case, myc overexpression represses miR-23, which normally binds to the 5′-UTR of the glutaminase mRNA leading to its degradation [106]. Glutaminase is also allosterically regulated through phosphate, which is required for the formation of the catalytically active tetramer [107], but requires supraphysiological concentrations *in vitro*.

### Protein biosynthesis and degradation

**Figure 6.**
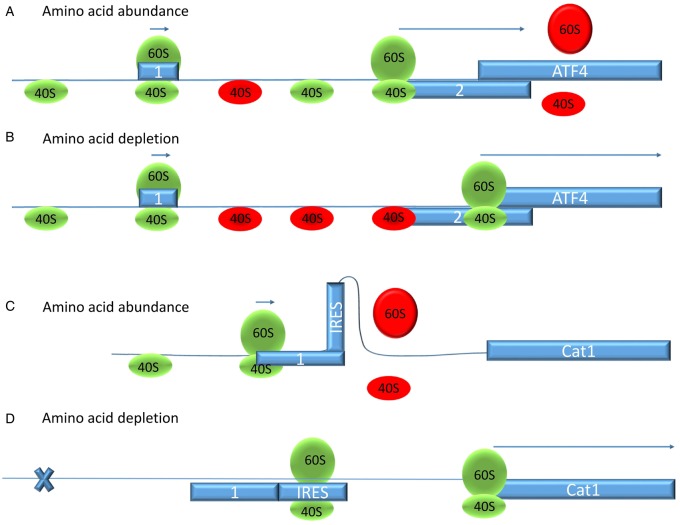
Stress-induced translation of specific mRNAs. Stress-induced translation makes use of upstream regulatory sequences preceding the start codon. The mRNA of transcription factor ATF4, which co-ordinates transcription of a variety of genes involved in stress responses, has two uORFs, numbered 1 and 2 in the figure. Under normal conditions (**A**), translation starts at the short uORF1 and the ribosome disassembles at the stop codon. The 40S subunit remains on the mRNA and reinitiates at uORF2 if levels of eIF2α·GTP·Met-tRNA_i_^Met^ are sufficient (ribosome green), which is incompatible with translation of ATF4. If levels of eIF2α·GTP·Met-tRNA_i_^Met^ are low (**B**), reinitiation is delayed (ribosome red), allowing the complex to reassemble at the more distant correct start codon for ATF4 translation. An alternative mechanism makes use of uORFs to change the secondary structure of mRNA (**C and D**). When translation is fast (**C**), uORF1 is translated, while the main ORF for the cationic amino acid transporter cat-1 is rarely translated. When cap-dependent translation is rare due to low levels of eIF2α·GTP·Met-tRNA_i_^Met^, the mRNA secondary structure changes, exposing an internal ribosome entry site (IRES). This is used to translate cat-1 more frequently (**D**).

Protein biosynthesis and degradation are major factors in amino acid homeostasis and are therefore tightly regulated by amino acid availability (Figure 2) [108–111]. To initiate translation, GTP-bound eIF2 must form a complex with the initiator methionine-tRNA. Together with other initiation factors and the 40S ribosomal subunit, a 43S pre-initiation complex is formed. This complex will bind to the 5′-cap structure of conventionally translated mRNAs with the aid of the eIF4F complex, comprising eIF4(A,G,E). Translation can be switched off through the sequestration of eIF4E by the 4E-BP1. This sequestration is regulated by phosphorylation of 4E-BP1, which when phosphorylated is unable to bind eIF4E. The 4E-BP1 protein is a target of the serine/threonine kinase mTORC1. Protein kinase complex mTORC1 not only regulates translation initiation through phosphorylation of 4E-BP1, but also through its downstream kinase p70S6K1, which in turn phosphorylates eIF4B and PDCD4 [112]. PDCD4 binds in its non-phosphorylated state to eIF4A and EIF4G, thereby inhibiting binding of mRNA [113]. When the 43S pre-initiation complex recognises the start codon AUG, eIF2α-bound GTP is hydrolysed to GDP and GDP-eiF2α is released to allow assembly of the full ribosome. When translation is ‘on’, eIF2α is recycled into its GTP-bound form through binding to the guanine nucleotide exchange protein eIF2B. Phosphorylation of GDP-eIF2α at position serine 51 by protein kinases, such as GCN2 and PERK, inhibits the exchange from GDP-bound eIF2α to GTP-bound eIF2α by interfering with its interaction with the guanine nucleotide exchange protein eIF2B [114]. As a result, eIF2α remains GDP-bound and is not available for the formation of the eIF2α·GTP·Met-tRNA_i_^Met^ complex, thereby switching translation ‘off’. These protein kinases are activated during cellular stress, for instance protein-folding problems in the ER (PERK) or lack of amino acids in the cytosol (GCN2) [27]. Thus, a variety of stimuli reduce translation, for instance lack of amino acids (via mTORC1) imbalances of the amino acid composition (via GCN2) and overload of protein folding (via PERK). At the same time, selected transcripts are translated more actively, the resulting proteins ameliorating amino acid starvation or imbalance. As a hallmark, these transcripts typically have two short ORFs upstream (uORF) of the start codon of the designated protein (Figure 6) [109,115]. The main transcription factor, which co-ordinates the responses to amino acid starvation, is ATF4 and its mRNA has such a structure [116,117]. The second uORF overlaps with the ATF4 start codon and acts as a strong inhibitor of ATF4 translation. Mutation of its start codon increases ATF4 translation more than 30-fold. The first uORF has no particular effect until eIF2α is phosphorylated. When eIF2α·GTP·Met-tRNA_i_^Met^ availability is reduced due to eIF2α phosphorylation, translation of the ATF4 mRNA increases ∼10-fold. This is obviously less than translation from an uORF-free mRNA, but allows selective translation under stress conditions. The mechanism is illustrated in Figure 6A,B: under non-stressed conditions, the 40S ribosomal subunit scans along the mRNA until it reaches the uORF1 start codon followed by assembly of the full ribosome and starting translation with eIF2α·GTP·Met-tRNA_i_^Met^. The ribosome disassembles at the end of uORF1, but the 40S subunit remains on the mRNA and scans further. At uORF2, the ribosome reassembles and initiates translation. Due to mechanisms that are unclear, this translation will be terminated soon after. Thus, ATF4 itself will not be translated. At low levels of eIF2α·GTP·Met-tRNA_i_^Met^, the ribosome fails to reassemble at the start codon of uORF2 and keeps scanning until the ATF4 mRNA starts. This provides more time for eIF2α-GDP to be recycled into eIF2α-GTP. Extension of the distance between uORF1 and uORF2 reduces the stress-stimulating effect of uORF1. An alternative mechanism, observed in the case of the cationic amino acid transporter cat1 and the sodium-neutral amino acid transporter SNAT2, involves cap-independent translation starting at an internal ribosome entry site (IRES, Figure 6C,D) [42,118]. In this mechanism, normal cap-dependent translation of uORFs favours an mRNA structure where the IRES is inaccessible. Owing to the secondary structure, translation of the main ORF is rare. When cap-dependent translation initiation is slow under stress conditions, an mRNA secondary structure is favoured that exposes the IRES, which is then used for translation of the main ORF (Figure 6C,D). The precise mechanism varies from gene to gene [119], but allows enhanced translation under stress conditions, such as amino acid depletion.

Upstream ORFs and ATF4-binding sites are found in many genes, but for the purposes of this review genes that carry out biosynthesis of non-essential amino acids (ASNS, alanine aminotransferase 2, PSAT, serine hydroxymethyltransferase 2, pyrroline-5-carboxylate reductase and glutamate-oxaloacetate transaminase) and genes encoding amino acid transporters (cat1, ASCT1, ASCT2, SNAT2, SNAT7, LAT1, EAAT5 and xCT) are notably abundant [83,120]. This is a particularly useful combination, because non-essential amino acids are important as an exchange substrate to bring in essential amino acids into the cytosol.

*Protein degradation:* Two main mechanisms are involved in protein digestion, namely the lysosome–autophagy system and the proteasome–ubiquitin system [121]. Autophagy (more specifically macroautophagy) is the process by which cellular compartments, such as membranes and organelles and also protein aggregates, are recycled back into their building blocks [122]. Microautophagy refers to the recycling of individual proteins. During macroautophagy, cells form double membrane-bound vesicles, autophagosomes, that sequester organelles, proteins or portions of the cytoplasm for delivery to the lysosome [123]. The sequestered contents are degraded in the lysosome, allowing cells to eliminate damaged or harmful components through catabolism and recycling, to maintain nutrient and energy homeostasis [124]. The process is constitutive, but is up-regulated under amino acid restriction through mTORC1 signalling [125–127]. In nutrient-sufficient conditions, mTORC1 interacts with a complex that contains ULK1 and Atg13 amongst other proteins. Upon mTORC1 inhibition, for example by amino acid depletion, mTORC1 dissociates from the ULK complex, leading to dephosphorylation of specific residues within ULK1 and Atg13, which are normally phosphorylated by mTORC1 [123]. Nutrient depletion is the most potent known physiological inducer of autophagy. In the majority of cultured mammalian cells, nutrient depletion induces autophagy within minutes, with maximal levels observed when cells are cultured in the simultaneous absence of nutrients and growth factors [128]. In mice, following starvation for 24–48 h, cells in most tissues display increased numbers of autophagosomes [129]. After fusion of autophagosomes with lysosomes, the protein content is digested by a variety of proteases, called cathepsins [130].

**Figure 7.**
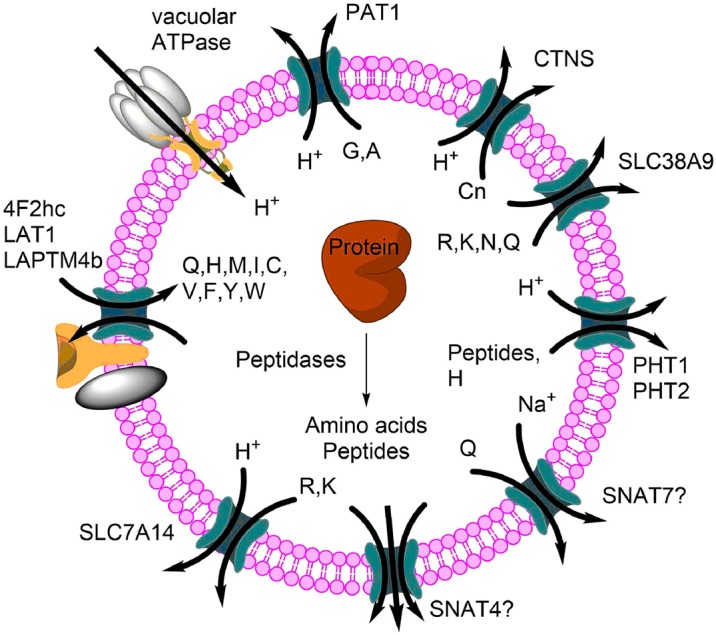
Lysosomal amino acid and peptide transport. Lysosomes are acidified through the action of the vacuolar proton-ATPase. This proton gradient is used to extrude amino acids through proton amino acid co-transporters (PATs) or peptide/histidine proton co-transporters (PHT1/SLC15A4 and PHT2/SLC15A3). Other amino acids can transfer in both directions through exchangers. SLC38A9 is the main lysosomal arginine sensor for mTORC1. SNAT4 and SNAT7 are putative (indicated by ?) lysosomal transporters.

As shown above, transporters play a key role in cellular amino acid homeostasis [131]. In contrast with cytosolic amino acid accumulation, it appears likely that lysosomes use a leak and harmoniser model (Figure 7). In this model, amino acids and peptides are constantly generated through proteolysis. Certain groups of amino acids and peptides are released through proton symporters. An overlap in substrate selectivity with amino acid antiporters would allow the net efflux of all amino acids over time. In support of this model, radiolabelled amino acids added to the outside of cells will quickly appear inside lysosomes [17]. Proteomic analysis [18,132] and immunofluorescence experiments have identified many transporters in lysosomes [131]. Typically, lysosomal transporters are proton co-transporters, allowing the extrusion of substrates using the proton electrochemical gradient. However, lysosomes contain sufficient Na^+^ [133] to support Na^+^ co-transporters, pH-optimum permitting. Owing to the inside-positive membrane potential, cations are extruded, even without proton co-transport. Transporters that occur both in the plasma membrane and in lysosomes will have the extracellular face of the transporter facing the inside of the lysosomes, due to the topology of vesicle formation [134]. For instance, proton amino acid transporter 1 (PAT1) mediates uptake of small neutral amino acids in the intestine [135], but mediates release of amino acids from the lysosomes [17]. LAT1, an amino acid exchanger found normally in the plasma membrane together with its 4F2hc subunit, was also identified on lysosomal membranes, to which it is targeted with the help of lysosomal protein LAPTM4b [18]. It appears likely that efflux of amino acids from lysosomes is rate-limiting to maintain significant amino acid levels within the lysosome for mTORC1 activation [136].

While the lysosome degrades large complexes including proteins, the controlled degradation of individual proteins is mediated by the proteasome [121,137]. Proteins are earmarked for recycling of amino acids by ubiquitination using a system of ubiquitin ligases. The protein is unfolded and de-ubiquitinated before hydrolysis into peptides of 8–10 amino acids [138]. These are either used as antigenic peptides or further digested into individual amino acids by cytosolic peptidases such as the thimet oligopeptidase [139]. Complete inhibition of the proteasome in NIH3T3 cells reduced the amino acid content of asparagine, aspartate and cysteine without affecting other amino acids [140]. These amino acids were absent from the media used in the experiment because they are not essential. Supplementation of cysteine, but not asparagine, restored viability of the cells. Enhanced expression of proteasomal protein, accordingly, increased cytosolic amino acid concentrations [141]. Increased protein biosynthesis, upon mTORC1 activation, is accompanied by increased synthesis of proteasomal proteins to cope with increased protein folding and misfolding [142].

### Integration

**Figure 8.**
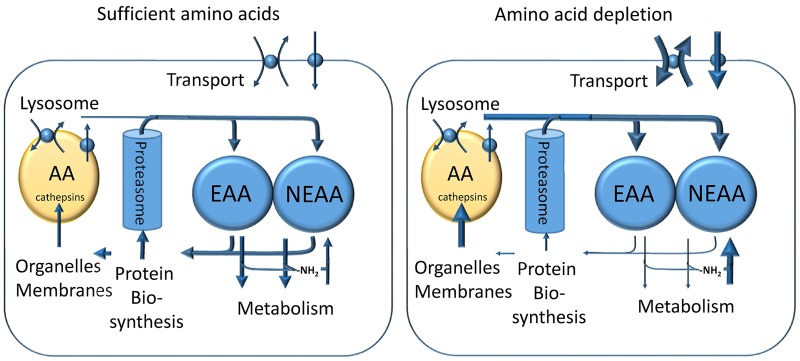
Cellular processes involved in amino acid homeostasis. Under nutrient-sufficient conditions, cells have a constant turnover of proteins that recycles most amino acids (AA) over time. Cellular protein forms a major storage polymer of amino acids. Net loss is mainly due to amino acid oxidation in the mitochondria, which is compensated through uptake of amino acids and/or biosynthesis. Homeostasis is achieved through exchange of essential amino acids (EAAs) with non-essential amino acids (NEAAs) and the transfer of amino groups from oxidised amino acids to amino acid biosynthesis. This homeostatic condition is maintained through an active mTORC1 complex. Under conditions of amino acid depletion, mTORC1 is inactivated. This increases the breakdown of cellular proteins through autophagy and reduces protein biosynthesis. The GCN2/ATF4 pathway may be activated in addition, resulting in transcription of genes involved in amino acid transport and biosynthesis of non-essential amino acids. These are used to import essential amino acids. Metabolism is autoregulated to minimise oxidation of amino acids.

The mechanisms outlined above can be integrated into a general model of cellular amino acid homeostasis (Figure 8): under nutrient-sufficient conditions, cells have a constant turnover of proteins that recycles most amino acids over time. Cellular protein forms a major storage polymer of amino acids. Net loss is mainly due to amino acid oxidation in the mitochondria, which is compensated through uptake of amino acids and/or biosynthesis. Homeostasis is achieved through exchange of essential amino acids with non-essential amino acids and the transfer of amino groups from oxidised amino acids to amino acid biosynthesis. This homeostatic condition is maintained through an active mTORC1 complex. Under conditions of amino acid depletion, mTORC1 is inactivated. This increases the breakdown of cellular proteins through autophagy and reduces protein biosynthesis. The GCN2/ATF4 pathway may be activated in addition, resulting in transcription of genes involved in amino acid transport and biosynthesis of non-essential amino acids. These are used to import essential amino acids. Metabolism is autoregulated to minimise oxidation of amino acids.

## Systemic amino acid homeostasis

Systemic amino acid homeostasis — defined here as the control of plasma amino acid levels — follows similar principles as cellular homeostasis, but functions are separated between organs: (i) entry (intestine) and reabsorption (kidney) through peptide and amino acid transporters; (ii) amino acid biosynthesis and degradation (all organs, urea cycle in liver); (iii) protein biosynthesis and degradation (mainly muscle) and (iv) regulation of protein intake and metabolism by the CNS and metabolic hormones.

These mechanisms are tightly regulated, resulting in plasma amino acid levels that are remarkably constant, even under conditions of protein restriction [143]. For instance, when rats were fed a 6 or 24% protein diet for 7–10 days, plasma amino levels were found to be essentially the same with the exception of glycine and serine, which doubled under protein restriction [144]. Liver amino acid levels are also held largely constant, with the exception of alanine, which is used in gluconeogenesis [145]. In mice, reduced intestinal absorption of amino acids or peptides also did not reduce or even increased plasma amino acid levels [146,147]. In humans, a similarly tight regulation of amino acid levels was observed [148]. Only on a high protein diet (1.5 g/kg body weight) was a postprandial increase in plasma amino acids observed [149]. Short-term fasting does not reduce plasma amino acid levels and even long-term fasting causes only modest changes [148]. Long-term malnutrition, however, causes significant reduction in plasma amino acid levels as observed in severe cases of Kwashiorkor [150].

An adult human needs to replace unavoidable losses of 20–25 g of protein/day [151]. The total protein turnover is much higher, ∼240 g per day. It is important to note that the energy content of protein is the same as that of carbohydrates (16–17 kJ/g), implying that nutrient amino acids are metabolised almost completely to CO_2_ and H_2_O when exceeding replacement of unavoidable protein losses.

### Entry and exit through amino acid and peptide transporters

**Figure 9.**
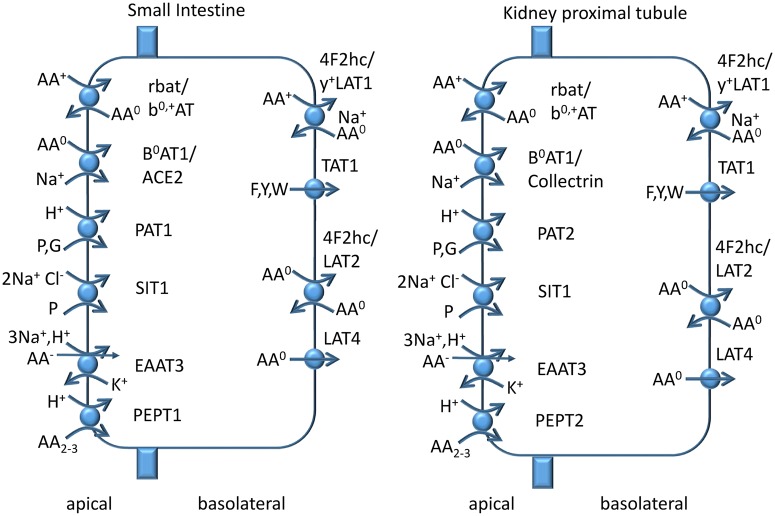
Epithelial amino acid transport. The apical and basolateral membrane of epithelial cells is endowed with different sets of transporters. Apical transporters actively translocate amino acids and peptides from the lumen of the intestine or of the proximal tubulus into the cytosol. Efflux is mediated by a mixture of uniporters and antiporters. Additional transporters (not shown) mediate the uptake of amino acids from blood plasma when required.

The breakdown of protein is mediated initially by gastric and pancreatic peptidases resulting in oligopeptides. These are further digested by membrane-anchored brush-border peptidases [152]. The final products of protein digestion are amino acids and di- and tri-peptides [153]. Epithelial amino acid transporters are different from amino acid transporters in non-polarised cells (Figure 9) [154]. The relevant amino acid transporters are listed in Table 1. In the apical membrane of the intestine, separate transporters are found for anionic amino acids (EAAT3) [155], cationic amino acids (the heteromeric transporter rbat/b^0,+^AT) [156], neutral amino acids (the heteromeric transporter B^0^AT1/angiotensin-converting enzyme 2) [157] and proline and glycine (PAT1 and SIT1) [135,158]; and di- and tri-peptides are transported by PEPT1 [159]. All of these transporters accumulate substrate inside the cell through various mechanisms. B^0^AT1, EAAT3 and SIT1 use the Na^+^-electrochemical gradient as a driving force with different stoichiometries (Table 1). PEPT1 and PAT1 are proton co-transporters that exploit the low extracellular pH in the intestine. Finally, b^0,+^AT is an amino acid exchanger that takes up cationic amino acids in exchange for neutral amino acids. Mouse models deficient for some of these transporters show signs of protein restriction, but plasma amino acid levels are normal [146,147]. This does not mean that reduced intestinal absorption does not have an impact on amino acid homeostasis, as it is compensated by reduced oxidation of amino acids. Reduced oxidation of amino acids can be monitored through urea production, which is proportional to amino acid oxidation.

To complete intestinal absorption, amino acids are released through the basolateral membrane. Basolateral transporters are different from those in the apical membrane [160]. These are designed to maintain intracellular amino acid levels when food is absent, but at the same time allowing a net efflux out of the cell. Similar to the principles described for cellular amino acid homeostasis above, antiporters are abundant in the basolateral membrane, such as 4F2hc-LAT2 and 4F2hc-y^+^LAT1 [161–163]. They allow the efflux of various ratios of amino acids as they occur in different food sources. Net efflux only occurs when intracellular amino acid concentrations exceed those in the blood stream and is mediated by TAT1 for aromatic amino acids [164] and most probably by LAT4 for other neutral amino acids [165]. Net efflux of cationic amino acids occurs through y^+^LAT1, but requires the uptake of plasma neutral amino acids in exchange [166]. Net efflux of anionic amino acids across the basolateral membrane has not been resolved.

Postprandial amino acid and peptide absorption result in increased plasma amino acid concentrations, although these are only significant at high protein loads [149,167]. It appears that plasma amino acid concentrations can briefly increase through intake, but that fasting does not lead to decreases in plasma amino acid levels with the exception of alanine, which is used for gluconeogenesis [148].

The limited effect of nutrient intake on plasma amino acid levels is caused by the liver, which metabolises ∼60% of all amino acids of a normal diet [143]. However, the extent to which amino acids are digested by the liver differs with alanine and glutamine [168] being well metabolised, while BCAAs and glutamate are barely removed by the liver [143]. Thus, many amino acids pass through to reach other organs. The kidney generates an ultrafiltrate of blood plasma in the glomeruli of the nephrons. The ultrafiltrate comprises all small molecules plus smaller amounts of proteins and peptides up to a molecular mass of 60 kDa. Apart from molecules that are excreted as waste products, such as urea and creatinine, all valuable metabolites and ions are reabsorbed in the proximal tubule [154,169]. The mechanisms of reabsorption are similar to those in the intestine, but may involve transporter variants (Figure 9). In the apical membrane of the human proximal tubule, separate transporters are found for anionic amino acids (EAAT3 [170] and rbat/AGT [171]), cationic amino acids (the heteromeric transporter rbat/b^0,+^AT) [156], neutral amino acids (the heteromeric transporter B^0^AT1/collectrin) [172] and proline and glycine (PAT2 and SIT1) [154,173]; di- and tri-peptides are transported by PEPT2 [174]. Mutations of apical renal transporters in humans have very little impact on plasma amino acid levels, but do result in significant loss of amino acids into the urine, which is used to diagnose the mutated transporter [175]. The loss is compensated through reduced amino acid oxidation [146].

Muscle has a similar combination of Na^+^-dependent amino acid symporters, uniporters and antiporters as described above for the amino acid homeostasis in cancer cells [176]. This provides a pool of amino acids at concentrations that are higher than in blood plasma for protein biosynthesis [55]. A notable difference is a thus far unidentified glutamine transporter that allows bidirectional transport depending on the metabolic state of the muscle (see below) [177]. Moreover, muscle cells express SNAT3 [178]. This transporter mediates Na^+^-dependent but electroneutral glutamine transport, due to a H^+^ antiport mechanism [179]. In conjunction with electrogenic Na^+^-glutamine co-transporters such as SNAT2, a mechanism could be proposed that would allow efflux or uptake depending on the metabolic state. Owing to the different ion stoichiometry, SNAT2 accumulates its substrate ∼100-fold, while SNAT3 accumulates only 10-fold. Thus, if SNAT2 is more abundant than SNAT3, amino acids would be taken up by muscle, and if SNAT3 dominates over SNAT2, efflux would occur. This mechanism has not been tested, but it is known that SNAT2 is highly regulated in muscle through insulin and mTORC1 [180–182], resulting in translocation to the cell surface. A similar mechanism could account for alanine release as a source for gluconeogenesis, but has not been investigated either.

The blood–brain barrier has a complex array of amino acid transporters that are important for amino acid homeostasis in the brain [183], but overall amino acid oxidation by the brain has no measurable effect on systemic amino acid homeostasis. Homeostatic levels of plasma amino acids appear to be particularly important for brain function as illustrated by many metabolic disorders (see below).

### Biosynthesis and degradation of amino acids

Organs contribute in different ways to amino acid homeostasis. The small intestine metabolises large amounts of glutamine and arginine, while releasing alanine, glycine and citrulline [184,185]. The liver consumes large amounts of alanine and releases glutamate [186]. Alanine is one of the major precursors for gluconeogenesis and is released in significant amounts from muscle in the early hours of fasting [187]. The release of alanine from muscle also increases during exercise [188]. The heart also releases alanine. Adipose tissue is similar to muscle tissue in releasing glutamine and alanine into the circulation [189]. Kidneys release significant amounts of serine into the circulation, and also convert phenylalanine into tyrosine, thereby producing the majority of this amino acid [184]. The kidneys also convert citrulline into arginine. Together with the intestine, this constitutes an interorgan shuttle that regulates arginine levels [185,190]. The brain generates its energy almost entirely from glucose. Uptake of amino acids is only required to replace small amounts of metabolised amino acids, which cannot be detected in arteriovenous differences [191]. The brain does, however, release measurable amounts of glutamine [192], demonstrating oxidative metabolism of amino acids, particularly BCAAs.

To use amino acids as fuels, the resulting amino groups or ammonia has to be disposed of as urea by the liver. However, amino acids are used as fuel by many organs and as a result amino groups are released either as ammonia or amino acids. As shown above, alanine, serine and glutamine are typically released by many tissues and transfer nitrogen to the liver. Consequently, increased protein intake initiates two major responses: first, an increase in protein synthesis in muscle driven by insulin (see below); second, an increased production of urea, allowing the use of amino acids as metabolic fuels [193]. Free ammonia from the circulation or locally produced ammonia, generated via the phosphate-activated glutaminase reaction, is used to synthesise carbamoylphosphate, which provides the first nitrogen for urea biosynthesis [194]. Not surprisingly, urea cycle activity is regulated by amino acids. *N*-acetyl-glutamate is an essential cofactor of carbamoylphosphate synthetase. Hepatic glutamate levels rise quickly after a meal or after injection of amino acids, and with it so do the levels of *N*-acetyl-glutamate. *N*-acetyl-glutamate in turn increases carbamoylphosphate synthetase activity [47]. Hepatic glutamate is largely generated through transamination from nutritional amino acids, rather than by glutamate transport into hepatocytes, which is very limited [195]. This mechanism allows the use of a wide variety of amino acids as fuels. Arginine is an allosteric activator of *N*-acetyl-glutamate synthetase [194]; however, *in vivo* this effect appears to be limited [47]. Leucine, in contrast, has a significant effect on urea generation through allosteric activation of GDH [48]. In the liver, this promotes the generation of glutamate, because glutamate is not taken up from the circulation, thereby fixing more ammonia when amino acids are used as energy metabolites in other tissues. Through transamination, amino groups of incoming amino acids are transferred onto aspartate, which provides the second nitrogen for urea biosynthesis. Thus, increased amounts of leucine increase glutamate production in the liver, which in turn activates urea synthesis [196]. Urea synthesis is also increased during fasting when alanine becomes the main precursor for gluconeogenesis. While most of the regulation of urea cycle activity resides within the liver [194], the intestine reduces amino acid oxidation after nutrient intake through the conversion of glutamine and arginine into citrulline. This reduces the activation of *N*-acetyl-glutamate synthetase, thereby allowing more amino acids to pass the liver [185].

Metabolism appears to have the greatest impact on plasma amino acid levels. Disruption of the mitochondrial branched-chain aminotransferase (BCATm) in mice elevated levels of leucine, isoleucine and valine 25-, 33- and 37-fold, respectively [197]. No other disturbance of amino acid homeostasis has similar effects. Rare disorders of amino acid metabolism provide further evidence for a central role of metabolism in keeping plasma amino acid levels at homeostatic concentrations [198]. In fact, many metabolic disorders are diagnosed through the elevation of amino acids, such as disorders of phenylalanine metabolism (phenylalanine elevated, [199]), tyrosine metabolism (tyrosine elevated, [200]), the metabolism of sulphur amino acids (methionine elevated, [201]), urea cycle disorders and related diseases, (glutamine elevated, arginine depleted, [202]), disorders of glycine, serine and proline metabolism (glycine elevated, serine depleted because mutations affecting synthetic pathways, proline elevated, [203]) and the metabolism of BCAAs elevated [204]. Many of these inherited disorders have severe consequences for CNS function, demonstrating a particular sensitivity of this organ to imbalances of amino acid homeostasis.

### Protein biosynthesis and degradation

Muscle is the largest reservoir of amino acids in higher organisms. Studies in rodents and humans have shown that protein synthesis in muscle after fasting is stimulated by re-feeding [205]. Later studies showed that this process is dependent on amino acids, particularly leucine [110,206]. Leucine was shown to activate mTORC1, but only in the presence of insulin [110]. This is in agreement with the notion that mTORC1 requires activation by growth factors to be susceptible to amino acid sensing [10]. The coincidence of postprandial amino acid absorption and insulin secretion increases muscle protein biosynthesis. In conjunction with an increase in protein biosynthesis, amino acid transport into muscle is up-regulated as well [181,207,208]. In addition to insulin, protein biosynthesis in muscle is also up-regulated by androgens and IGF-1 (insulin-like growth factor 1), with androgens stimulating IGF-1 release [209]. However, these processes are typically slow, regulating protein synthesis over many days, and are therefore unlikely to have a direct impact on plasma amino acid levels [209]. Protein biosynthesis by the liver is an important contributor to body protein synthesis and is also critical for the maintenance of the plasma oncotic pressure. Synthesis of albumin is regulated by amino acids and declines rapidly under conditions of amino acid depletion to spare amino acids [210].

Autophagy plays a significant role in the maintenance of plasma amino acid concentrations under starvation conditions. Autophagy is up-regulated immediately after birth when the placental nutrient supply is cut off. Mice lacking atg5, which is essential for autophagosome formation, have plasma amino acid levels that are 20% lower than wild-type mice [211]. Amino acids in turn regulate systemic autophagy, most prominently in the liver, but less so in muscle where insulin appears to be the main regulator [212].

### Regulation of systemic amino acid homeostasis by hormones and the CNS

As outlined above, amino acids are the most relevant regulators of their own homeostasis, particularly within cells. However, some endocrine hormones play a significant role in systemic amino acid homeostasis.

Insulin and IGF-1 promote incorporation of amino acids into protein, particularly in muscle [213–215]. Hyperinsulinaemia was found to decrease plasma leucine concentration and to inhibit protein breakdown and leucine oxidation. Insulin also regulates amino acid uptake into muscle [216]. Insulin and growth factor signalling are mediated through their canonical signal transduction pathways involving tyrosine kinase receptors, PI3-kinase and protein kinase Akt. Protein kinase Akt then phosphorylates and inactivates TSC2 and PRAS40, both of which are negative regulators of the mTORC1 complex [215]. Growth hormone also stimulates protein biosynthesis, by reducing ureagenesis and converting glutamine in the liver into glutamate, which is released back into the circulation instead of generating urea [217].

Insulin regulates amino acid metabolism, but vice versa amino acids also regulate insulin release in β-cells of the pancreas. Leucine activates GDH, increasing the metabolism of glutamine into 2-oxoglutarate and subsequent TCA cycle intermediates. This increases mitochondrial ATP production, which, together with ATP derived from glucose metabolism, closes K_ATP_ channels. The resulting membrane depolarisation increases cytosolic calcium ion levels and triggers release of insulin-containing vesicles [218]. Enhancement of insulin release by leucine has an inbuilt negative feedback mechanism. GDH is negatively regulated by GTP, which is produced by the succinyl-CoA synthetase reaction in the TCA cycle. As a result, mitochondrial ATP production from glutamine is self-limiting. Evidence for this mechanism has been gained from mutations in GDH that cause insensitivity to allosteric regulation by GTP. This mutations cause hypersecretion of insulin and hypoglycaemia after a protein-rich meal [219]. Insulin release is regulated also by other amino acids. GPR142 appears to underlie the insulinotropic effects of food-derived tryptophan and its effect on the secretion of intestinal hormones (incretins) that stimulate insulin release [34]. GPR142 signals through Gαq and is expressed in enteroendocrine cells of the small intestine, particular in K cells, which secrete the incretin GIP (gastric inhibitory peptide). GPR142 is also found in β-cells, where it may increase cytosolic calcium levels [34]. Accordingly, heterologous expression of GPR142 shows an inositol-3-phosphate response to tryptophan and phenylalanine. Oral administration of tryptophan strongly increased GIP secretion, which was not observed in GPR142-deficient mice. Insulin secretion was significantly reduced in GPR142-deficient mice. Elevated levels of BCAAs have been noted in individuals with metabolic syndrome and appear to precede the onset of diabetes [220]. These results can be interpreted as a sign of increasing insulin resistance, not only affecting the deposition of glucose, but also affecting the metabolism of BCAAs, for instance in muscle [221–223].

Glucagon increases gluconeogenesis, which particularly during early fasting is supported by amino acids. Accordingly, the total amino acid concentration in blood plasma decreases during glucagon excess with plasma levels of alanine, citrulline, proline, ornithine, tyrosine, glycine and threonine falling most significantly. Urea nitrogen also increased, suggesting that glucagon may indirectly regulate amino acid levels through regulation of gluconeogenesis [224]. In agreement with this notion, glucagon also increases transcription of Sodium-neutral amino acid transporter SNAT2 in hepatocytes through a cAMP response element in the promoter region [225].

Glucocorticoids are released upon stress and nutrient limitation and inhibit protein translation. At the same time, glucocorticoids increase the release of alanine from muscle, the main final product of protein degradation in this tissue [226]. Inhibition of protein synthesis is thought to occur at the step of translation initiation. Through the action of the glucocorticoid receptor, transcription of REDD1 (regulated in development and DNA damage) is increased. This causes reduced phosphorylation of 4E-BP1 and p70S6K1, thereby inhibiting protein translation. The actions of REDD1 are thought to be mediated through the mTORC1 complex [227].

Fibroblast growth factor 21 (FGF21) is now recognised as a metabolic hormone that is produced upon protein restriction [146,228,229]. FGF21 is a significant regulator of fat metabolism. In protein metabolism, it has been suggested as a secretagogue for the exocrine pancreas, thereby facilitating protein digestion [230]. FGF21 is an atypical growth factor, the signalling of which requires β-klotho as a co-receptor [231].

Enteroendocrine cells of the intestine produce a variety of hormones that are secreted upon nutrient intake, such as GLP-1 (glucagon-like peptide 1) and GIP [232]. These hormones prepare the body for nutrient intake by enhancing insulin secretion and reducing appetite, but are not directly involved in amino acid homeostasis. PYY is probably the most protein-specific gastrointestinal hormone. It is produced by L-cells and its production reacts to the protein content of the diet. PYY-deficient mice are hyperphagic and become obese as a consequence. More importantly, the satiating effects of high protein diets were abolished in *PYY* null mice [233].

Food intake is tightly regulated through processes in the CNS [234,235]. Appetite is regulated in the arcuate hypothalamic nucleus and there is evidence that leucine levels and mTORC1 signalling contribute to modulation of the relevant pathways, resulting in appetite suppression [235]. Growth of cells and organisms is largely dependent on increasing protein mass. Indeed, it has been proposed that food intake in many organisms is regulated by the protein requirement of the growing body [236]. In mature organisms, the protein requirement is, however, small. A normal human adult, for instance, requires 8000 kJ/day. A Western diet typically comprises 50% carbohydrate, 15% protein and 35% fat. This would equate to 70 g of protein, which is about three times the unavoidable daily loss of protein. Protein malnutrition is only observed during general malnutrition or when the diet is largely based on carbohydrate-rich staples, such as rice or potatoes. The brain has developed very sensitive mechanisms to detect amino acid imbalances [237]. For instance, rodents strictly avoid diets that are deficient in a particular essential amino acid [238]. Sensing essential amino acid depletion occurs in the anterior piriform cortex (APC) via GCN2 activation and subsequent glutamatergic signalling to influence behaviour. Mapping of the APC output during essential amino acid insufficiency shows axons projecting to the hypothalamus as well as other regions that are involved in feeding and locomotion. Marginal essential amino acid deficiency in the form of methionine restriction promotes hyperphagia similar to low-protein diets [235].

### Integration

**Figure 10.**
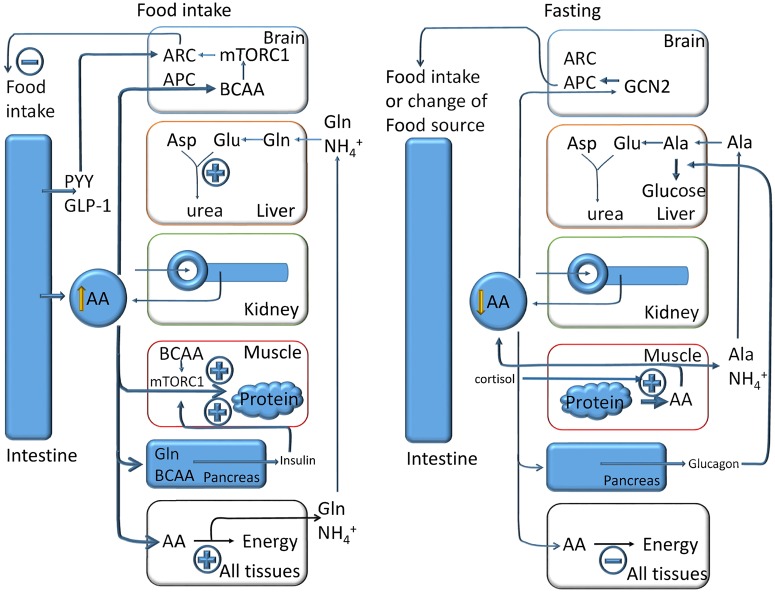
Systemic amino acid homeostasis. After food intake, gastrointestinal hormones are secreted that suppress appetite. The plasma amino acid pool increases, which contributes to insulin release from the pancreas. Insulin increases transport of amino acids into muscle and their incorporation into protein. The remaining amino acids are metabolised. Amino groups are delivered as glutamine to the liver, where they are together with ammonia used to generate urea. During fasting, amino acid oxidation is reduced. Muscle protein is degraded to replete the amino acid pool if necessary. Alanine is released from muscle to synthesise glucose. Amino acid imbalance or restriction will activate GCN2 signalling in the anterior piriform cortex (APC) to change the source of food. ARC, arcuate hypothalamic nucleus.

Plasma amino acid levels are tightly regulated (Figure 10). Food intake briefly increases plasma amino acid levels, which causes insulin release and mTORC1-dependent protein synthesis in muscle. Amino acid uptake into muscle is up-regulated. Excess amino acids are oxidised by most tissues, resulting in increased urea production. Increase in amino acid oxidation appears to be largely regulated through allosteric mechanisms. Mutations in amino acid metabolising enzymes cause significant increases in the corresponding plasma amino acid levels. Short-term fasting does not result in depletion of plasma amino acids due to reduced protein synthesis and the onset of autophagy. Glucocorticoids are recognised as hormones that reduce protein synthesis under stress conditions. Due to the fact that half of all amino acids are essential, reduction in protein synthesis and amino acid oxidation are the only two measures to reduce amino acid demand. Long-term malnutrition thus results in depletion of plasma amino acids. The CNS appears to generate a protein-specific response upon amino acid depletion, resulting in avoidance of the inadequate diet. High protein levels, in contrast, contribute together with other nutrients to a reduction in food intake.

## Abbreviations

4E-BP1: 4E-binding protein 1
4F2hc-LAT1: Glycoprotein-associated large neutral amino acid transporter 1
AKT (acronym) APC: anterior piriform cortex
ASCT: alanine-serine-cysteine transporter
ASNS: asparagine synthetase
ATF4: activating transcription factor 4
atg13: autophagy 13
BCAAs: branched-chain amino acids
BCKAs: branched-chain keto-acids
BCKD: branched-chain keto-acid dehydrogenase
CARE: CCAAT/enhancer-binding protein (C/EBP)-ATF response element
CHOP: C/EBP homologous protein
CNS: central nervous system
CPS1: carbamoylphosphate synthetase
EAAT3: excitatory amino acid transporter 3
eIF2α: eukaryotic initiation factor 2α
ER: endoplasmic reticulum
ERK: extracellular signal-regulated kinase
FGF21: fibroblast growth factor 21
GAP: GTPase-activating protein
GATOR (acronym) GCN2: general control non-derepressable 2
GDH: glutamate dehydrogenase
GIP: gastric inhibitory peptide
GPRC6: G-protein coupled receptor family member C6
IGF-1: insulin-like growth factor 1
IRES 4-Beta: mTORC mechanistic target of rapamycin complex
PAT1: proton amino acid transporter 1
PAT1/4: proton amino acid transporters 1 and 4
PDCD: programmed cell death
PEPT1: peptide transporter 1
PRAS40: Proline-rich AKT substrate 40 kDa
PSAT1: phosphoserine aminotransferase
REDD1: regulated in development and DNA damage
Rheb: Ras homologue enriched in brain
SARS: seryl-tRNA synthetase
SIT1: system imino transporter 1
SLC: solute carrier
SNAT: sodium-neutral amino acid transporter
TAT1: T-type amino acid transporter
T1R: taste receptor member 1
TCA: tricarboxylic acid
TSC2: Tuberous sclerosis complex 2
ulk1: UNC51-like kinase 1
uORF: upstream open reading frames
uORFs: upstream open reading frames
